# Association Between Relative Fat Mass and Risk of Cognitive Impairment: The Role of Social Determinants of Health

**DOI:** 10.62641/aep.v54i2.2121

**Published:** 2026-04-15

**Authors:** Dongxi Wang, Huan Liang, Zhe Gan, Jie Zhang, Junli Wu

**Affiliations:** ^1^Medical College, Wuhan University of Science and Technology, 430065 Wuhan, Hubei, China; ^2^Department of Cadre Ward I, General Hospital of Central Theater Command, 430070 Wuhan, Hubei, China

**Keywords:** cognitive impairment, obesity, relative fat mass, social determinants of health, longitudinal studies

## Abstract

**Objective:**

This study examined the relationship between relative fat mass (RFM) and the risk of new-onset cognitive impairment and tested the mediating effect of social determinants of health (SDOH).

**Methods:**

Data originated from the China Health and Retirement Longitudinal Study. Data from 6147 participants without cognitive impairment at baseline were included. RFM was calculated and categorised into quartiles, whereas a cumulative SDOH score was constructed and grouped into tertiles. The association between RFM and new-onset cognitive impairment was assessed by using Kaplan–Meier curves, multivariable Cox proportional hazards models and restricted cubic splines (with piecewise regression for threshold analysis). Subgroup and joint effect analyses were performed on SDOH and RFM.

**Results:**

During a mean follow-up of 7.16 years, 1242 incident cases of cognitive impairment occurred. Elevated RFM was a significant risk factor for cognitive impairment (hazard ratio [HR] = 1.024, 95% confidence interval [CI]: 1.015–1.033, *p* < 0.001). This correlation was nonlinear, and RFM was estimated to have an inflection point of 26.45. The analysis of interaction effects showed that the risk of cognitive impairment in the population at risk (low SDOH/obesity) was higher by 91% (HR = 1.908, 95% CI: 1.516–2.401, *p *< 0.001) relative to that in the high-SDOH/nonobesity group. Notably, amongst obese individuals, high SDOH/obesity was not associated with a significantly increased risk (HR = 1.047, 95% CI: 0.828–1.325, *p* = 0.701).

**Conclusions:**

Elevated RFM is significantly associated with an increased risk of cognitive impairment. However, this relationship is moderated by socioeconomic context. Low SDOH is a serious aggravating factor for the risk of high RFM, whereas high SDOH may play a massive buffering and protective role. Intervention approaches should be designed by accounting for personal metabolic factors along with the social environment, with particular attention paid to dually disadvantaged groups to avoid cognitive deterioration.

## Introduction

The burden of cognitive impairment continues to rise as population ageing accelerates [1]. Meanwhile, as a pervasive public health issue affecting large populations, obesity has been consistently associated with declines in global cognition and across multiple cognitive domains [2, 3, 4]. Although the body mass index (BMI) remains the standard metric for defining obesity, it is still inadequate in its ability to reflect body fat distribution [5]. Relative fat mass (RFM) is a measure based on the product of waist circumference and height. Compared with BMI, it shows a stronger correlation with body fat percentage measured by dual-energy X-ray absorptiometry. In addition, RFM is highly effective in forecasting health conditions, including metabolic syndrome and cardiovascular diseases‎ [6, 7]. Recent prospective studies have stated that high levels of central obesity are strongly related to cognitive deterioration and raised the possibility of dementia, with the implication that body fat distribution characteristics can be a key contributor to cognitive impairment pathogenesis [8, 9]. However, prospective evidence linking RFM to incident cognitive impairment remains scarce amongst Asian older adults, and the hazardous cutoff and dose–response pattern of RFM have yet to be clearly established.

Beyond physiological mechanisms, disparities in the social environment may also influence cognitive health [10]. Social determinants of health (SDOH) are deemed to be crucial factors in society that form the root cause of population health inequity. The concept of SDOH refers to the situations in which individuals are born, grow, live, work and age, as well as the social forms and forces that predetermine these situations [11]. These determinants have an immense effect on health behaviours and on the management of chronic diseases and cognitive functions [12]. Populations with poor social health often face a high risk of cognitive impairment. For example, a study on a sample of indigenous women revealed that low income and educational levels presented a highly significant relationship with cognitive deterioration [13]. However, whether inequalities in SDOH supplement the adverse effects of obesity on cognitive function remains unclear.

In contrast to previous cross-sectional studies, our study uses a nationally representative longitudinal cohort in China to evaluate the association between RFM and the risk of new-onset cognitive impairment, identify potential inflection points and examine the modifying role of SDOH. Our findings aim to inform the development of stratified and precision-oriented strategies for preventing cognitive decline.

## Subjects and Methods

### Study Population

We sourced the data used in this study from the 2011 and 2020 China Health and Retirement Longitudinal Study (CHARLS) surveys. CHARLS uses stratified and multistage probability sampling design and initiates follow-up surveys after every 2–3 years [14]. The study protocol was approved by the Peking University Institutional Review Board (IRB00001052-11015), and all participants provided written informed consent in accordance with the ethical principles of the Helsinki Declaration [15]. Four waves of CHARLS data (2011–2020) were used. At the 2011 baseline, 17,708 community-dwelling respondents with age ≥45 years were successfully interviewed. We sequentially excluded the following: (1) 6654 individuals with missing cognitive tests; (2) 1945 individuals with missing or out-of-range height/waist measurements; (3) 1110 individuals with missing SDOH variables; and (4) 1852 individuals who had already been diagnosed with cognitive impairment or memory-related diseases at baseline. We retained 6147 participants for the final analyses.

### Calculation of RFM

In this study, we calculated RFM by using a previously validated formula [6]: RFM = 64 – (20 × height/waist circumference) + (12 × sex). Height and waist circumference were measured on-site in centimetres, divided by 100 to convert into metres and then used in the literature-derived formula for calculation. Sex was coded as 0 for male and 1 for female. A high RFM value indicates a high body fat content. RFM was treated as an estimate of body fat percentage (unit: %) [6]. We firstly divided the continuous RFM into quartiles (Q1–Q4), used Q1 as the reference and presented the dose–response relationship (trend test) and event rates across RFM levels in the Results section. For our subsequent joint analyses, we adopted the clinically common high body fat cut-offs (men ≥25%, women ≥35%) and reclassified participants into high/nonhigh RFM groups to examine whether the exposure (low SDOH) exerts a strong amplification effect on cognitive risk amongst individuals with high adiposity and to align with the existing literature on body composition. Notably, these specific cut-off values are primarily based on empirical standards for body fat percentage, and no unified guideline for the Chinese population exists. Therefore, the findings related to this stratification should be interpreted as exploratory analyses based on RFM [6, 16].

### Assessment of SDOH

By drawing on the U.S. Healthy People 2030 agenda and Braveman’s framework, we constructed an SDOH score covering five domains: education access and quality, economic stability, health-care access and quality, neighbourhood and built environment and social and community context [12, 17]. The selection and operational definitions of specific items were guided by established SDOH frameworks but tailored to the available variables in the CHARLS database to reflect exposure across these core dimensions optimally [18]. A favourable condition was coded 1, and an unfavourable condition was coded 0 (Table 1). The eight items were summed to yield an individual SDOH score ranging from 0 to 8, with high values indicating advantageous social environments. Participants were classified into low (1–4), medium (5) and high (6–8) groups on the basis of cohort-specific tertile cut-offs derived from the distribution of scores in our study population to balance data-driven distributions with clinical utility.

**Table 1.** **Assessment of SDOH**.

Variable	Coding description
Educational attainment	0 = no formal education; 1 = primary education or above
Employment status	0 = unemployed; 1 = employed or retired
Household consumption expenditure	0 = below median; 1 = above median
Health insurance coverage	0 = no coverage; 1 = covered
Access to primary health-care facilities	0 = not available; 1 = available
Housing quality/housing condition	0 = other; 1 = reinforced concrete structure
Marital status	0 = not currently married (single/divorced/widowed); 1 = married
Social participation in the past month	0 = no participation; 1 = participation

SDOH, social determinants of health.

### Assessment of Cognitive Impairment

We assessed cognitive function in the CHARLS cohort by using a structured cognitive test battery. This battery included temporal orientation (identifying the current year, month, date, day of the week and season); serial subtraction (continuously subtracting 7 from 100); immediate and delayed recall of a 10-word list; and a figure drawing task. The overall cognitive score is 0–31, with high scores denoting improvement in cognitive functioning. The reliability and validity of the cognitive questionnaire used in the CHARLS surveys were evaluated by using Cronbach’s alpha and the Kaiser Meyer–Olkin test, respectively. These tests showed good internal consistency across all individuals, with an overall reliability of 0.85 and validity of 0.76 for the CHARLS 2011–2018 surveys [19]. Cognitive impairment was defined by using age-group-specific cut-offs. Participants were assigned to one of five age strata. Within each stratum, individuals whose score fell one standard deviation below the age-specific mean were classified as cognitively impaired. This age-adjusted criterion follows the standard described in the reference [20].

### Assessment of Covariates

Covariates were measured at baseline. They included the following: (1) Demographic information: age and place of residence. (2) Lifestyle information: smoking status and alcohol use. (3) History of disease: hypertension, dyslipidaemia, diabetes, heart disease, stroke and cancer. Given the inclusion of a sex-specific coefficient in the RFM formula, sex was not included as an independent covariate in the models to avoid multicollinearity but was instead treated as an inherent component of the RFM measure.

### Statistical Analysis

All analyses were performed with R 4.2.3 (R Foundation for Statistical Computing, Vienna, Austria) and Zstats 1.0 (available at: https://www.medsta.cn/software; Zstats Development Team, Beijing, China). Normality was examined by using the Shapiro–Wilk test and Q–Q plots. Normally distributed variables were presented as mean ± SD ($\bar{x}$ ± S) and compared amongst groups by using one-way ANOVA, whereas nonnormally distributed variables were expressed as median (P25, P75) and compared by employing the Mann–Whitney U or Kruskal–Wallis test as appropriate. Categorical data were displayed as frequencies and percentages, with comparisons between groups being performed with the chi-squared test. Kaplan–Meier curves were utilised to estimate the cumulative incidence of cognitive impairment in various RFM groups, and the overall difference was estimated by using the log-rank test. Cox proportional hazards models were employed to evaluate the longitudinal relationship between the risk of cognitive impairment and RFM. Nonlinear relationships were analysed by the use of a restricted cubic spline (RCS) model. The threshold effect analysis was then conducted by applying a piecewise Cox regression model, and the likelihood ratio test was performed to compare the goodness of fit between the linear and nonlinear models. Subgroup analyses were conducted to examine the potential modifying effects of sociodemographic characteristics and health status. All statistical tests were two sided, with *p* < 0.05 considered statistically significant. In the joint RFM–SDOH analysis, five pairwise comparisons were performed. Therefore, the Bonferroni correction was applied, and the adjusted significance level was set at α* = 0.01 (*p* < 0.01 was considered statistically significant). Covariates with missing values (all missing rates <1.5%) were imputed via multiple imputation by using the mice package in R, generating 20 datasets under the MCAR assumption. The specific variables, missing rates and imputation models are detailed in **Supplementary Table 1**. The procedure did not materially alter the distribution of any variable (all *p* > 0.05), as shown in the comparison before and after imputation (**Supplementary Table 2**). The final estimates for regression models were obtained by pooling the results from all imputed datasets in accordance with Rubin’s rules.

## Results

### Characteristics of the Study Population

We included 6147 participants, with a mean age of 57.67 ± 8.84 years, in this study. Of these participants, 3358 (54.7%) were male and 2789 (45.3%) were female. Significant differences (*p* < 0.05) were observed across the RFM quartile groups in terms of age, sex, residence place, smoking status, alcohol use, hypertension history, dyslipidaemia, diabetes, heart disease, cancer and SDOH levels. Details are presented in Table 2.

**Table 2.** **Baseline characteristics of participants**.

Characteristics		Total (n = 6147)	Q1 (n = 1535)	Q2 (n = 1538)	Q3 (n = 1535)	Q4 (n = 1539)	χ^2^/F	*p-*value
Age (years)		57.67 ± 8.84	58.94 ± 8.57	58.48 ± 8.87	55.57 ± 8.77	57.67 ± 8.76	44.6	<0.001
Sex								
	Female	2789 (45.3)	3 (0.2)	55 (3.6)	1192 (77.7)	1539 (100)	4846.323	<0.001
	Male	3358 (54.7)	1532 (99.8)	1483 (96.4)	343 (22.3)	0 (0.0)		
Residence								
	Rural	4612 (75.0)	1301 (84.8)	1142 (74.3)	1072 (69.8)	1097 (71.3)	111.636	<0.001
	Urban	1535 (25.0)	234 (15.2)	396 (25.7)	463 (30.2)	442 (28.7)		
Smoking								
	Yes	2688 (43.7)	1, 174 (76.5)	1, 079 (70.2)	318 (20.7)	117 (7.6)	2252.358	<0.001
	No	3459 (56.3)	361 (23.5)	459 (29.8)	1217 (79.3)	1422 (92.4)		
Alcohol use								
	Yes	2595 (42.2)	1013 (66.0)	1012 (65.8)	381 (24.8)	189 (12.3)	1462.191	<0.001
	No	3552 (57.8)	522 (34.0)	526 (34.2)	1154 (75.2)	1350 (87.7)		
Hypertension								
	Yes	1486 (24.2)	200 (13.0)	430 (28.0)	364 (23.7)	492 (32.0)	167.216	<0.001
	No	4661 (75.8)	1335 (87.0)	1108 (72.0)	1171 (76.3)	1047 (68.0)		
Hyperlipidaemia								
	Yes	628 (10.2)	69 (4.5)	165 (10.7)	169 (11.0)	225 (14.6)	88.804	<0.001
	No	5519 (89.8)	1166 (95.5)	1373 (89.3)	1366 (89.0)	1314 (85.4)		
Diabetes								
	Yes	365 (5.9)	31 (2.0)	94 (6.1)	95 (6.2)	145 (9.4)	75.895	<0.001
	No	5782 (94.1)	1504 (98.0)	1444 (93.9)	1440 (93.8)	1394 (90.6)		
Heart disease								
	Yes	708 (11.5)	108 (7.0)	161 (10.5)	188 (12.2)	251 (16.3)	67.392	<0.001
	No	5439 (88.5)	1427 (93.0)	1377 (89.5)	1347 (87.8)	1288 (83.7)		
Stroke								
	Yes	113 (1.8)	20 (1.3)	32 (2.1)	31 (2.0)	30 (1.9)	3.323	0.344
	No	6034 (98.2)	1515 (98.7)	1506 (97.9)	1504 (98.0)	1509 (98.1)		
Cancer								
	Yes	55 (0.9)	8 (0.5)	6 (0.4)	22 (1.4)	19 (1.2)	13.856	0.003
	No	6092 (99.1)	1527 (99.5)	1532 (99.6)	1513 (98.6)	1520 (98.8)		
SDOH								
	Low	1746 (28.4)	499 (32.5)	388 (25.2)	450 (29.3)	409 (26.6)	41.097	<0.001
	Medium	1814 (29.5)	486 (31.7)	460 (29.9)	425 (27.7)	443 (28.8)		
	High	2587 (42.1)	550 (35.8)	690 (44.9)	660 (43.0)	687 (44.6)		

**Note**: Data are presented as mean ± SD, median (P25, P75) or n (%). Q1–Q4 = 1st–4th quartiles of relative fat mass (RFM); SDOH, social determinants of health.

### Association Between RFM and Cognitive Impairment

#### Kaplan–Meier Curve Analysis of RFM and New-Onset Cognitive Impairment

**Fig. 1.**
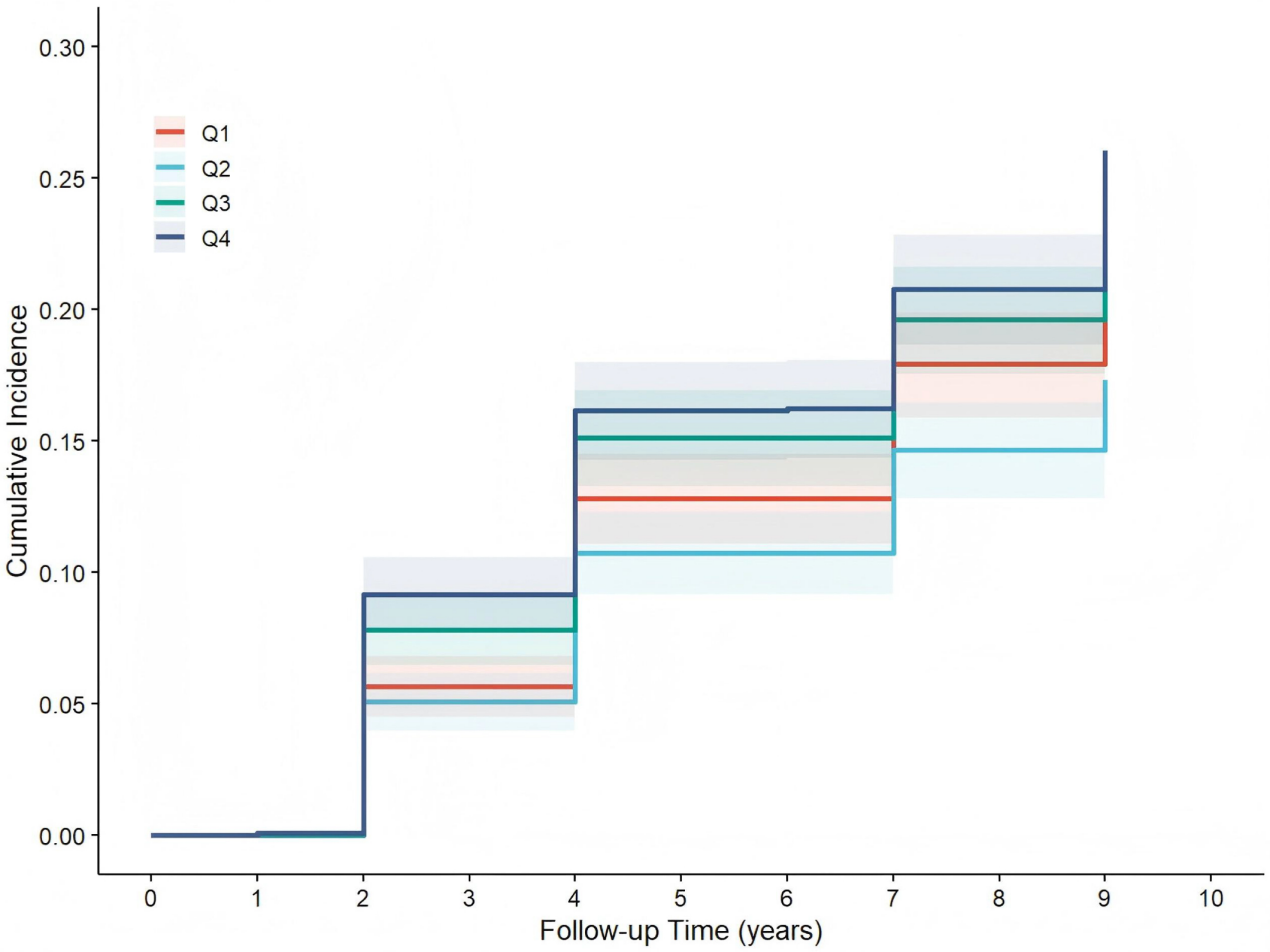
**Comparison of the cumulative incidence of cognitive impairment across RFM groups**. Note: Q1–Q4 = 1st–4th quartiles of relative fat mass (RFM).

During a mean follow-up of 7.16 years (interquartile range: 4–9 years; maximum: 9 years), 1242 incident cases of cognitive impairment (20.2%) were documented. Participants were stratified into quartiles based on RFM: Q1 (RFM 10.00–24.91, n = 1535) with 289 cases, Q2 (RFM 24.92–30.53, n = 1538) with 240 cases, Q3 (RFM 30.54–39.49, n = 1535) with 347 cases and Q4 (RFM 39.50–50.88, n = 1539) with 366 cases. Kaplan–Meier curve analysis revealed a progressive increase in the cumulative incidence of cognitive impairment over time across all groups. We observed a statistically significant difference in the cumulative incidence of new-onset cognitive impairment amongst the four RFM quartiles (χ^2^ = 36.581, *p* < 0.001), as shown in Fig. 1. The mean follow-up times were comparable across RFM quartiles (Q1: 7.11 years; Q2: 7.26 years; Q3: 7.17 years; Q4: 7.09 years), suggesting a balanced duration of observation amongst the comparison groups. We verified rhe proportional hazards assumption by using Schoenfeld residuals (global test *p* = 0.358; *p* for RFM groups = 0.713).

#### Multivariable Cox Regression Analysis of RFM and New-Onset Cognitive Impairment

We performed multivariable Cox regression analyses by using the occurrence of cognitive impairment (coded as yes = 1 and no = 0) as the dependent variable, RFM as a continuous variable and RFM quartiles (Q1–Q4) as independent variables. Three models were developed: Model 1 was unadjusted. Model 2 was adjusted for age, residence place (coded as urban = 1 and rural = 2) and SDOH (coded as low = 1, medium = 2 and high = 3). Model 3 was further adjusted for smoking (coded as no = 1 and yes = 2), alcohol use (coded as no = 1 and yes = 2), hypertension, diabetes, hyperlipidaemia, stroke, cancer and heart disease (all coded as no = 1 and yes = 2). The results from all models consistently demonstrated that a high RFM was a significant risk factor for cognitive impairment. Furthermore, a significant increasing trend in the risk of cognitive impairment was observed with ascending RFM levels (*p*_for trend_ < 0.001). The detailed results are presented in Table 3.

**Table 3.** **Multivariable Cox regression analysis of RFM and incident cognitive impairment**.

Groups		Model 1		Model 2		Model 3	
		HR (95% CI)	*p*-value	HR (95% CI)	*p*-value	HR (95% CI)	*p*-value
RFM continuous		1.016 (1.009–1.022)	<0.001	1.022 (1.014–1.028)	<0.001	1.024 (1.015–1.033)	<0.001
RFM categories							
	Q1	Ref.		Ref.		Ref.	
	Q2	0.816 (0.688–0.968)	0.002	0.907 (0.764–1.077)	0.265	0.926 (0.779–1.101)	0.382
	Q3	1.196 (1.023–1.398)	0.025	1.405 (1.199–1.646)	<0.001	1.497 (1.248–1.797)	<0.001
	Q4	1.271 (1.089–1.483)	0.002	1.455 (1.246–1.699)	<0.001	1.574 (1.296–1.911)	<0.001
*p*_for trend_			<0.001		<0.001		<0.001

**Note**: Model 1 adjusts for none. Model 2 adjusts for age, residence and SDOH. Model 3 adjusts for: age, residence, SDOH, smoking, alcohol use, hypertension, hyperlipidaemia, diabetes, heart disease, stroke and cancer. Sex was excluded because it is already embedded in the RFM formula, leading to structural collinearity. HR, hazard ratio; CI, confidence interval.

#### RCS Analysis of the Association Between RFM and Incident Cognitive Impairment

**Fig. 2.**
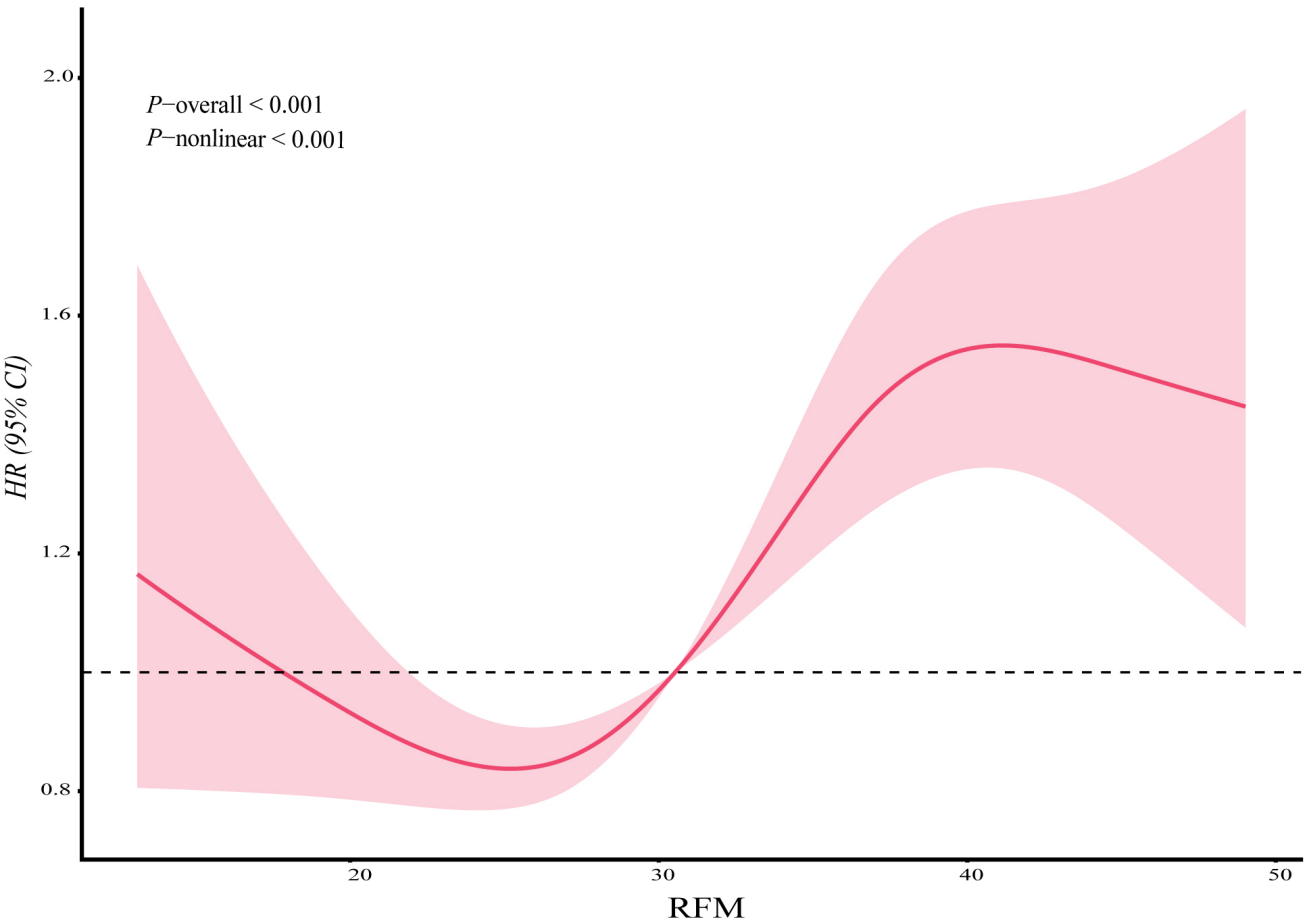
**Dose–response relationship between RFM and incidence of cognitive impairment**. Note: RFM, relative fat mass; HR, hazard ratio; CI, confidence interval.

We employed an RCS model to investigate the RFM–cognitive impairment link further. The results, depicted in Fig. 2, revealed a significant nonlinear relationship. This relationship was supported by the test for the overall association (*p*-overall < 0.001) and the test for nonlinearity (*p*-nonlinear < 0.001).

#### Threshold Effect Analysis of RFM on Incident Cognitive Impairment

The piecewise Cox regression model identified a significant threshold effect in the association between RFM and the risk of cognitive impairment (*p* for the likelihood ratio test = 0.036). When RFM was bel ow the inflection point of 26.45, the association with cognitive impairment risk was not significant (hazard ratio [HR] = 0.987, 95% confidence interval [CI]: 0.952–1.023, *p* = 0.466). By contrast, above the threshold of 26.45, each one-unit increase in RFM was associated with an approximately 2.8% increased risk of cognitive impairment (HR = 1.028, 95% CI: 1.014–1.042, *p* < 0.001). The detailed results are presented in Table 4.

**Table 4.** **Threshold effect and piecewise regression results of RFM**.

Outcome		HR (95% CI)	*p*-value
Model 1 Fitting model by standard linear regression		1.023 (1.014–1.032)	<0.001
Model 2 Fitting model by two-piecewise linear regression			
Inflection point (RFM)		26.45	
	<26.45	0.987 (0.952–1.023)	0.466
	≥26.45	1.028 (1.014–1.042)	<0.001
	*p* for likelihood test		0.036

**Note**: RFM, relative fat mass; HR, hazard ratio; CI, confidence interval.

#### Subgroup Analysis of the Association Between RFM and Incident Cognitive Impairment

**Fig. 3.**
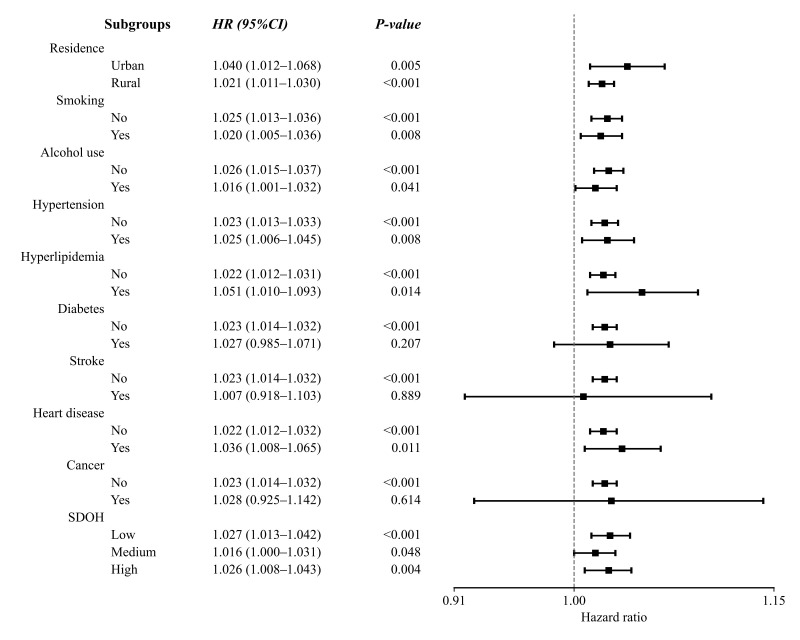
**Subgroup analysis of the association between RFM and cognitive impairment risk**. Note: SDOH, social determinants of health; HR, hazard ratio; CI, confidence interval.

Subgroup analyses showed that in most strata defined by residence, smoking, alcohol use and multimorbidity, high RFM was significantly associated with an increased risk of incident cognitive impairment (*p* < 0.05). However, amongst participants with diabetes, stroke, or cancer, the 95% CIs of HRs all crossed 1, indicating no statistically significant link between RFM and cognitive impairment risk in these specific disease subgroups (*p* > 0.05). Furthermore, a positive association between increasing RFM and elevated cognitive impairment risk was consistently observed across all SDOH strata. Notably, the highest risk estimate was identified in the low-SDOH group (HR = 1.027, 95% CI: 1.013–1.042, *p* < 0.001), as illustrated in Fig. 3.

#### Joint Association of RFM and SDOH With the Risk of Cognitive Impairment

**Fig. 4.**
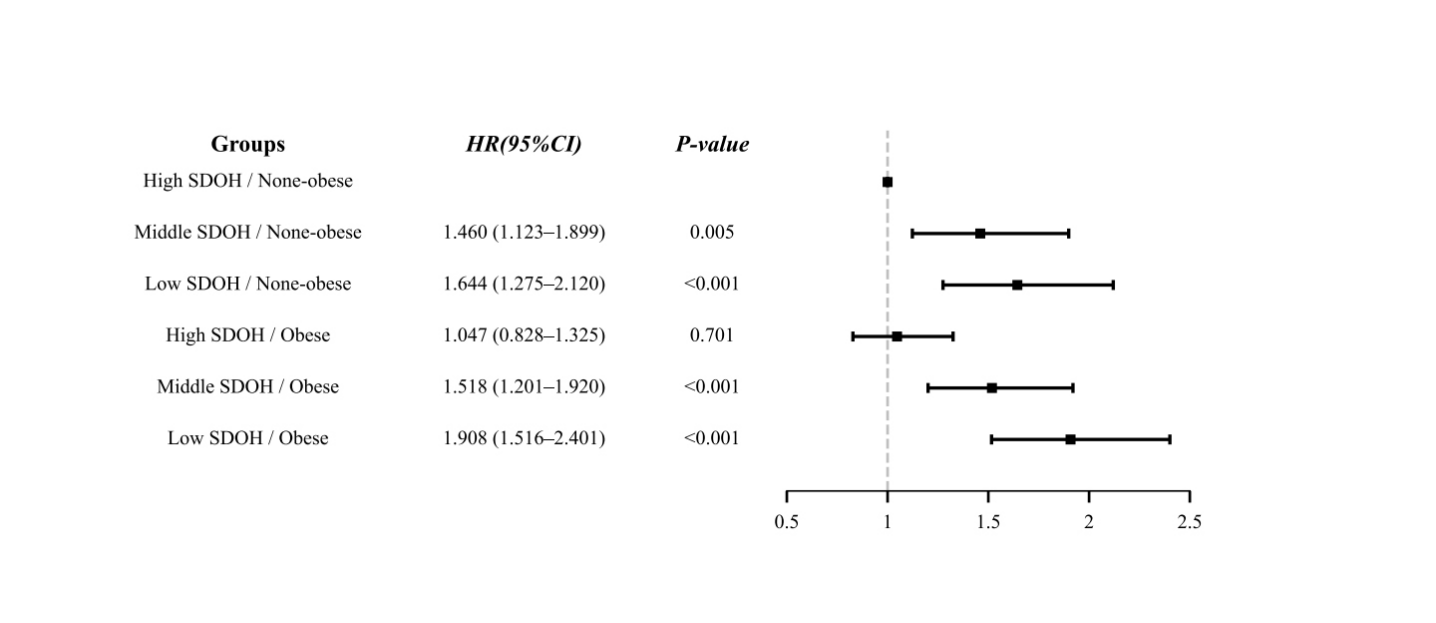
**Joint effect analysis of SDOH and RFM on the risk of cognitive impairment**. Note: SDOH, social determinants of health; HR, hazard ratio; CI, confidence interval. The five joint groups were compared with the high-SDOH/nonobese reference group. A Bonferroni-corrected significance level of *p* < 0.01 was applied for these comparisons. The reported *p*-values are unadjusted.

The analysis of interaction effects did not reveal a significant interaction between SDOH and RFM (*p*_for interaction_ = 0.434). In the joint analysis using the high-SDOH/nonobese group as the reference, the risk of cognitive impairment was found to be significantly elevated in several groups, with a 46% increase in the middle-SDOH/nonobese group (HR = 1.460, 95% CI: 1.123–1.899, *p* = 0.005), a 64% increase in the low-SDOH/nonobese group (HR = 1.644, 95% CI: 1.275–2.120, *p* < 0.001), a 52% increase in the middle-SDOH/obese group (HR = 1.518, 95% CI: 1.201–1.920, *p* < 0.001) and a 91% increase in the low-SDOH/obese group (HR = 1.908, 95% CI: 1.516–2.401, *p* < 0.001). Notably, the high-SDOH/obese group showed no statistically significant difference from the reference (HR = 1.047, 95% CI: 0.828–1.325, *p* = 0.701). These results are shown in detail in Fig. 4, where the *p* values shown are uncorrected, and statistical significance was set as Bonferroni-adjusted *p* < 0.01.

## Discussion

Our observational investigation, which is based on the national representative cohort CHARLS, demonstrates that a nuanced relationship exists between RFM and the risk of new-onset cognitive impairment. Moreover, it assesses the possibility of the modifying effect of SDOH on this relationship. This study suggests that high levels of RFM are typically related to the increased risk of cognitive impairment. However, this relationship is not exclusive but is influenced by socioenvironmental background. Joint analysis showed that an unfavourable SDOH profile significantly exacerbates the risk associated with high RFM, whereas a favourable SDOH profile appears to buffer it. Given the above situation, a crucial need exists to orchestrate biological measures with sociological indicators in risk stratification in population ageing.

Previous research suggests that excessive fat accumulation may affect cognitive function through multiple metabolic–neural pathways [3, 21]. Epidemiological analyses and Mendelian randomisation studies on Asian populations indicate that every 0.27 kg increase in visceral fat corresponds to an acceleration of cognitive ageing by approximately 0.7 years, implicating mechanisms, such as insulin resistance, chronic low-grade inflammation and impaired neurovascular coupling, in accelerating cognitive decline [22]. Furthermore, a randomised controlled trial found that for every 1% reduction in body weight following an 18-month lifestyle intervention in individuals with obesity, brain biological age reduced by an average of 8–9 months, suggesting that body fat control helps improve metabolic status and neural functional connectivity, thereby slowing cognitive ageing [23]. This body of evidence collectively emphasises the importance of obesity as a potential risk factor for cognitive impairment. Our study addresses a gap in such research based on Chinese populations and, consistent with international works, highlights the value of incorporating RFM into cognitive impairment risk assessment strategies [24]. Importantly, our study further explored the role of SDOH in the association between relative fat mass and cognitive impairment risk, indicating the necessity of including SDOH in stratified intervention strategies. The buffering effect observed in high-SDOH individuals may be explained by their increased access to resources that support healthy lifestyles and improved management of metabolic conditions, as well as by the cognitive reserve fostered through high education and social engagement, factors highlighted in prior studies on socioeconomic status (SES) and cognition [25, 26, 27, 28].

Another key finding is the nonlinear relationship between RFM and cognitive impairment risk, where an inflection point is identified at approximately RFM = 26.45, above which the risk increases sharply. This pattern closely mirrors the inverted U-shaped BMI–dementia curve reported by Kivimäki *et al*. [29] in a European cohort and the phased trajectory observed in a Korean longitudinal study [30], indicating that the effect of adiposity on brain health is not monotonic but rather confined to a dangerous fat zone. We propose that the directional change around the inflection point reflects a qualitative shift in fat-depot characteristics. The adipose tissue expandability hypothesis [31] states that when RFM <26%, excess lipids are preferentially stored subcutaneously; subcutaneous adipocytes secrete abundant adiponectin, enhancing hippocampal insulin signalling and synaptic plasticity via the AdipoR1–AMPK pathways [32]. Once total fat exceeds the above threshold, further weight gain is chiefly deposited viscerally, leading to high IL-6 and TNF-α levels, systemic insulin resistance, impaired IRS-1 tyrosine phosphorylation in the brain, reduced β-amyloid clearance and microvascular endothelial dysfunction [32, 33]. Interestingly, sex-stratified analyses showed that the independent linear association between RFM and cognitive impairment did not reach statistical significance within either sex. We interpret this result not as the absence of effects but as a consequence of the RFM formula incorporating sex and of women having physiologically higher RFM values than men. The observed nonlinearity partly reflects heterogeneity in sex distribution across the RFM range and sex-specific biological susceptibility: the upper RFM quartile is composed mainly of women, who already carry a higher baseline risk of cognitive impairment than men. Therefore, the effect of fat mass on cognition is jointly modulated by age, sex distribution and adipose tissue topography rather than by a simple linear relationship.

Subgroup analyses (by residence, lifestyle and chronic conditions) further confirmed the strength of this association across most population strata. Although this association was not statistically significant amongst individuals with diabetes or stroke history (potentially because of our limited sample size or confounding by the pathophysiology of these diseases themselves), the direction of the risk effect associated with high RFM remained consistent in most other subgroups. We found that the modifying effect of SDOH was quite significant. This finding is more important than generalisability across conventional subgroups. Despite the absence of a statistically significant interaction term, our joint analysis clearly revealed a buffering effect of social factors. In participants who had high SDOH, beneficial social resources appeared to counteract the harmful effect of high RFM and made the risk insignificant (HR = 1.047, *p* = 0.701). On the other hand, a significant correlation was found between high RFM and cognitive impairment in individuals with low SDOH (HR = 1.908). This result is an indication that a vulnerable background of social health may contribute to the negative consequences of metabolism and inflammatory events on the neurocognitive system that are caused by obesity [21, 25]. A cohort study on Finns [26] showed that a high educational level nullified the negative relationship between midlife high BMI and cardiovascular risks with later-life cognition. In the same way, SES and residential environment significantly influenced cognitive function [25, 27]. Long-term wealth disadvantage is positively correlated with cognitive decline, and high SES may not only reduce the risk of cognitive impairment but also enhance the potential for cognitive resilience [28]. The other dimensions of the SDOH, which are urban–rural environment, marital status, social engagement and health-care access, have also demonstrated different effects on cognitive functioning via diverse pathways [30, 34, 35, 36]. Collectively, these findings indicate that populations with low SDOH encounter overlapping disadvantages in social resources, education, health behaviours and body fat load; these intertwined social and physiological risk factors interact synergistically to create a dual burden, rendering such populations highly vulnerable to cognitive impairment.

Our study contributes to the existing literature in two key aspects. Firstly, it examined the longitudinal association between body fat and cognitive risk in a middle-aged and elderly Chinese population by using the RFM index, a metric that is closely aligned with adipose distribution, thereby supplementing evidence on adiposity and cognitive health from a Chinese national cohort. Secondly, through joint analysis, it quantified the modifying effect of social factors on the obesity–cognition relationship within a Chinese population, providing a reference for precise prevention.

Nevertheless, our research has a number of limitations. Firstly, the definition of obesity relied on empirical body fat percentage cutoffs (≥25% for men and ≥35% for women). While this approach facilitates international comparison, the lack of unified, population-specific diagnostic criteria for Chinese individuals may affect the precision of risk stratification. Future studies would benefit from establishing nationally representative cutoffs. Secondly, sex was not taken as an independent covariate in our multivariable Cox regression models. This decision was based on rigorous statistical diagnostics: given that the RFM formula contains a sex parameter (coefficient of 12), structural multicollinearity was observed between RFM and sex (VIF >5). Sex was considered as an inherent constituent of the composite RFM risk profile instead of an independent confounder to prevent the unstable estimation of the parameters. Although this approach does not give us a full opportunity to separate the independent effects of sex and body fat, it does not weaken the usefulness of RFM as a screening tool in identifying high-risk individuals. Thirdly, the SDOH score failed to cover deep factors, like social capital or perceived discrimination. Lastly, the determination of cognitive impairment depends on neuropsychological tests. Therefore, future studies should be complemented with multimodal verification associated with biomarkers.

## Conclusions

Elevated RFM effectively identifies individuals at elevated risk for cognitive impairment. However, the realisation of this risk is critically moderated by SDOH. Future prevention strategies must therefore integrate individual physiological risk (e.g., RFM) with socioenvironmental context into stratified interventions, moving beyond weight management alone. Meanwhile, future studies should be dedicated to establishing normal reference ranges and obesity diagnostic cutoffs for body fat percentage specific to the Chinese population, thereby furnishing clinical and public health practice with precise tools.

## Availability of Data and Materials

The datasets generated and/or analyzed during the current study are available in the CHARLS repository, http://charls.pku.edu.cn/.
