# Supplementary material for: Association Between Relative Fat Mass and Risk of Cognitive Impairment: The Role of Social Determinants of Health
Source: Actas Esp Psiquiatr. 2026 Apr 15;54(2):317–27. doi: 10.62641/aep.v54i2.2121 (PMC13180675; doi:10.62641/aep.v54i2.2121)
Supplement: Supplementary file 1 [file ActEsp-54-2-317-327-s1.zip › ActEsp-54-2-317-327-s1/Supplementary Table.docx]

**Supplementary Table 1** Missing rate, mechanism assumption, and imputation model for each variable.

| Variables | type | miss_pct | pattern | imp_model | imp_function |
| --- | --- | --- | --- | --- | --- |
| Hyperlipidemia | nominal | 1.48 | MCAR | Multinomial logit | mice::mice.impute.polyreg() |
| Diabetes | nominal | 0.63 | MCAR | Multinomial logit | mice::mice.impute.polyreg() |
| Heart disease | nominal | 0.50 | MCAR | Multinomial logit | mice::mice.impute.polyreg() |
| Hypertension | nominal | 0.37 | MCAR | Multinomial logit | mice::mice.impute.polyreg() |
| Cancer | nominal | 0.34 | MCAR | Multinomial logit | mice::mice.impute.polyreg() |
| Stroke | nominal | 0.16 | MCAR | Multinomial logit | mice::mice.impute.polyreg() |
| Residence | nominal | 0.03 | MCAR | Multinomial logit | mice::mice.impute.polyreg() |

**Note:** nominal, categorical variables.

Missing mechanism: MCAR , missing completely at random (Little’s test p≥0.05 or no association with observed covariates).

Imp_model abbreviations: Multinomial logit, multinomial logistic regression. Function names refer to specific mice imputation methods (polyreg for nominal).

**Supplementary Table 2** Baseline characteristics pre- and post-imputation.

| Variables | Pre-imputation | Post-imputation | *χ*^2^*/t* | *P* |
| --- | --- | --- | --- | --- |
| Hyperlipidemia | | | | |
| Yes | 611 (10.1%) | 628 (10.2%) | 0.054 | 0.816 |
| No | 5, 445 (89.9%) | 5, 519 (89.8%) |  |  |
| Diabetes | | | | |
| Yes | 365 (6.0%) | 365 (5.9%) | 0.008 | 0.929 |
| No | 5, 743 (94.0%) | 5, 782 (94.1%) |  |  |
| Heart disease | | | | |
| Yes | 706 (11.5%) | 708 (11.5%) | 0.002 | 0.964 |
| No | 5, 410 (88.5%) | 5, 439 (88.5%) |  |  |
| Hypertension | | | | |
| Yes | 1, 479 (24.2%) | 1, 486 (24.2%) | 0.001 | 0.976 |
| No | 4, 645 (75.8%) | 4, 661 (75.8%) |  |  |
| Cancer | | | | |
| Yes | 55 (0.9%) | 55 (0.9%) | 0.000 | 0.986 |
| No | 6, 071 (99.1%) | 6, 092 (99.1%) |  |  |
| Stroke | | | | |
| Yes | 113 (1.8%) | 113 (1.8%) | 0.000 | 0.990 |
| No | 6, 024 (98.2%) | 6, 034 (98.2%) |  |  |
| Residence | | | | |
| Rural | 4, 610 (75.0%) | 4, 612 (75.0%) | 0.000 | 0.992 |
| Urban | 1, 535 (25.0%) | 1, 535 (25.0%) |  |  |
